# The Yield of One vs. Two Blood Cultures in Children: Under-Detection and Over-Testing

**DOI:** 10.3390/antibiotics13020113

**Published:** 2024-01-23

**Authors:** Anat Zalmanovich, Elizabeth Temkin, Dikla Biran, Yehuda Carmeli

**Affiliations:** 1 National Institute for Antibiotic Resistance and Infection Control, Israel Ministry of Health, Tel Aviv 64239, Israel; 2 Tel Aviv Sourasky Medical Center, Tel Aviv 64239, Israel; 3 Faculty of Medicine, Tel Aviv University, Tel Aviv 6139001, Israel

**Keywords:** blood cultures, diagnostic stewardship, pediatrics

## Abstract

We aimed to determine whether obtaining two blood cultures (BCs) instead of one improved the detection of bloodstream infections (BSIs) in children. For this descriptive study, we used surveillance data collected in 2019–2021 from all Israeli hospitals serving children. The sample included 178,702 culturing episodes. One BC was taken in 90.1% of all episodes and 98.2% of episodes in the emergency department. A true pathogen was detected in 1687/160,964 (1.0%) of single-culture episodes and 1567/17,738 (8.9%) of two-culture episodes (*p* < 0.001). The yield was significantly different even when considering only the first BC in two-culture episodes: 1.0% vs. 7.5%. Among 1576 two-culture episodes that were positive for a true pathogen, the pathogen was detected only in the second culture in 252 (16.0%). We estimated that if a second culture had been taken in all episodes, an additional 343 BSIs by a true pathogen would have been detected. Among 1086 two-culture episodes with commensal bacteria, the second BC was sterile in 530 (48.8%), suggesting contamination. A commensal was isolated in 3094/4781 (64.7%) positive single-culture episodes, which could represent BSI or contamination. The yield of a single BC bottle was low, reflecting both lower sensitivity of a single bottle and the taking of single bottles in patients with a low probability of BSI.

## 1. Introduction

Bloodstream infection (BSI) in children is a medical condition with significant morbidity and mortality [1]. Timely pathogen identification and antibiotic sensitivity testing can optimize treatment, reduce mortality [2,3,4], and prevent unnecessary antibiotic use. Blood volume is the most critical factor in the recovery of a pathogen from blood cultures (BCs); however, obtaining an insufficient amount of blood is common [5]. In one study, 48% of BC bottles drawn from adults contained an inadequate amount of blood [6]. Another factor to be considered is intermittent bacteremia that may be missed by a single culture, even with adequate blood volume [5,7]. Thus, the number of BCs taken may increase detection of bacteremia, both by increasing the total blood volume drawn and by increasing opportunities to capture the pathogen. The frequency of intermittent bacteremia is not well described. In a study of *Staphylococcus aureus* bacteremia, intermittent bacteremia occurred in 13% of episodes [8].

Studies in adults have demonstrated that taking multiple BCs increases pathogen isolation, with the first BC set detecting approximately 75% of pathogens, the second set adding 15–20%, and the third set adding about 7% [9,10]. Guidelines recommend obtaining 2–4 BC sets from different venipunctures per blood culturing episode [11,12], and advise against taking a single set because of its low sensitivity and potential difficulties in the interpretation of results [13]. 

In children, the benefit of a second BC is less certain and only a few studies have been published, with mixed results [14,15,16,17]. The Infectious Disease Society of America guidelines recommend obtaining two BCs from children weighing more than 1 kg, with a recommended total blood volume of 2 mL for children weighing ≤1 kg, 4 mL for children weighing 1.1–2 kg, 6 mL if 2.1–12.7 kg, 20 mL if 12.8–36.3 kg, and 40–60 mL for children over 36.3 kg [12]. The maximum volume of pediatric BC bottles is 4 mL; thus, taking one BC is insufficient for all children weighing more than 2 kg. Taking more than one BC increases the likelihood of identifying bacteria in low-level and intermittent bacteremia [18] increases the likelihood of detecting fastidious bacteria specific to the pediatric population, such as *Kingella*, and enables clinicians to distinguish between contamination and BSI caused by a common commensal. Moreover, taking multiple BCs likely increases the total volume of blood sampled. 

Despite these guidelines, collecting multiple BCs from children is not universally practiced [7,14,19]. Reasons for taking only one BC include the belief, based on data from the 1970s, that children and especially neonates have higher bacterial loads and more continuous bacteremia than adults [20]. Since then, multiple studies have confirmed that low-level bacteremia is more common than previously thought, occurring in up to 60% of all pediatric patients with a positive BC [21], even in children younger than 2 months [22]. Other reasons for taking single BCs are the inconvenience, distress, lack of cooperation, need for specially trained staff to draw blood from a child, and the risk of exacerbating anemia in critically ill children [5]. These difficulties in obtaining multiple BCs from children, weighed against the importance of detecting BSI, make it imperative to examine the benefit of taking more than one BC in the pediatric population. We aimed to determine whether obtaining two BCs instead of one improved detection of BSI in children.

## 2. Results

All 28 hospitals that serve children contributed data (n = 669 hospital-months). A total of 178,702 blood culturing episodes were documented in 131,068 children in 158,028 hospital admissions. The median age was 1 year (IQR: 1 month–3 years) and 45% were female. Pediatric bottles (able to contain a maximum of 4 mL of blood) were used in 88.9% (158,815/178,702) of all episodes. A single BC was taken in 91% of episodes. More than half of single BC episodes occurred in children older than 1 year; for them, the blood volume in a single pediatric bottle would not meet requirements. 

Table 1 shows the distribution of pathogens detected in single-culture vs. two-culture episodes. The most common pathogens in single-culture episodes were *E. coli* (11.3%) and *S. aureus* (11.2%), while the most common pathogens in two-culture episodes were *K. pneumoniae* (15.5%) and *E. coli* (13.8%).

Table 2 shows the characteristics associated with taking more than one BC per episode. Taking more than one BC was most common in the PICU (30.0%) and least common in the ED (1.8%). Taking more than one BC was also more common among children aged 12–18 (18.6%) and afterday 3 of hospitalization (35.7%).

A true pathogen was detected in 1.8% (3263/178,702) of all episodes. A true pathogen was detected in 1.0% (1687/160,964) of single-culture episodes, compared to 8.9% (1576/17,738) of two-culture episodes (*p* < 0.001). The difference in detection rate was present in all wards but was most pronounced in the ED (Table 3). There, a true pathogen was detected in only 0.8% (685/85,213) of episodes with one BC vs. 12.4% (199/1604) of episodes with more than one BC. The difference in detection rate was still significant when considering only the first draw: a true pathogen was detected on the first culture in 7.5% (1324/17,738) of two-culture episodes (*p* < 0.001 for comparison to 1.0% of single-culture episodes.

A true pathogen was detected in 1576 episodes in which two cultures were taken; 1324 were detected by the first BC, and an additional 252 were detected only by the second BC. Thus, taking a second culture increased the yield of detecting BSI by a true pathogen by 19.0%. The increase in yield increased with age: 11.7% in age < 1 month, 20.2% in ages 1–12 months, 21.7% in ages 1–11 years, and 24.8% in ages 12–18. 

Using the average of 19% increased detection of BSI by taking a second BC, we estimated that drawing only one BC missed 343 BSIs caused by a true pathogen. Indeed, we found that, in 577 cases of single-culture episodes testing negative for a true pathogen, a true pathogen was detected in the subsequent 7 days.

A commensal bacterium was detected in 2.4% of episodes that were negative for a true pathogen. Among the 1086 two-culture episodes with commensal bacteria detected in the first culture, the second culture was sterile in 530 (48.8%), indicating that the first sample would be classified as contaminated by NHSN surveillance criteria. A commensal was isolated in 3094 single-culture episodes (representing 64.7% of all positive episodes), leaving uncertainty as to whether they represent contamination or BSI.

## 3. Discussion

We compared the yield of one vs. two BCs for detecting BSI in children in a large dataset of all Israeli hospitals with a pediatrics division. We found that only one culture was taken in 90.1% of pediatric blood culturing episodes. Pediatric BC bottles containing a maximum of 4 mL of blood were used in 88.9% of the cases, representing a lower-than-recommended blood volume for children older than 1 year. Among two-culture episodes, taking a second culture increased pathogen detection by 19.0%, and increased with age. The pathogen detection rate in single-culture episodes was very low compared to two-culture episodes (1.0% vs. 8.9%), especially in the ED (0.8% vs. 12.4%). Nevertheless, more than half (51.7%) of all BSIs were detected in single-culture episodes. We estimated that by drawing only one BC, 343 BSIs went undetected, representing 10.5% of the detected BSIs during the study period.

Previous studies of the value of collecting a second culture in children have had mixed results. Among those showing a benefit was a study of pediatric cancer patients in which BSI detection increased from 12% to 23% when two cultures were taken instead of one [15]. Similarly, a study of neonates in India found that 16% of BSIs would have been missed had a second culture not been taken [16]. Tran et al. evaluated a decision support intervention that increased the percentage of obtaining two-culture per episode from 12% (similar to our findings) to 88%. Detection of a true pathogen rose from 3.7% to 7.3%, which the authors attributed both to the additional bottles and collection of a larger blood volume [14]. In contrast, a smaller study of 20 neonates with positive BCs reported no added benefit of a second BC [17].

We found a marked difference in the pathogen detection rate between episodes with one versus two BCs taken, even when considering only the results of the first of two BCs (1.0% vs. 7.5%). This difference probably stems from the practice of reflexively taking a single BC in patients with a low probability of BSI; it is likely that the majority of single BCs taken were unwarranted. The higher detection rate among two-culture episodes suggests that clinicians are able to identify patients at high risk of BSI and draw two BCs in those cases. Better strategies for patient selection are needed to minimize unnecessary BC collection and to maximize BSI identification.

Specific guidance on when to draw BCs is limited. A few BSI prediction tools have been proposed (and rarely implemented in clinical practice), mostly in adults [23]. For example, based on a sample of adult ED patients, Shapiro et al. recommended taking BC in patients with at least one major criterion (e.g., temperature > 39.5°C) or two minor criteria (e.g., age > 65 years, hypotension). This tool had high sensitivity (97%) but poor specificity (29%) [24]. The need for BC can also be determined according to clinical syndrome. BCs are recommended in syndromes with a high pretest probability of BSI (e.g., meningitis, septic shock) and discouraged in syndromes with a low pretest probability of BSI (e.g., non-severe community-acquired pneumonia, cystitis) [25,26,27]. Parikh et al. studied over 5000 children presenting to the ED with a diagnosis of asthma, bronchitis, skin infection, or community-acquired pneumonia [28]. None of these conditions warrants a BC, but a BC was collected in 21% of patients. BC positivity was 0%, 0%, 1%, and 2%, respectively.

In addition to higher yield, another reason to take two BCs is to differentiate between contamination and BSI caused by a common commensal. According to NHSN definitions, commensals must be isolated on two separate occasions to meet criteria for BSI [29]. In our study, the second culture was sterile in 48.8% of two-culture episodes with commensal bacteria in the first culture, suggesting contamination rather than BSI. Commensals made up the majority of organisms isolated in single-culture episodes (64.7%); in these cases, BSI cannot be ruled in or ruled out. This ambiguity has important clinical consequences, as detection of commensals may lead to prolonged hospital stays, higher costs, and unnecessary antibiotic use [30,31]. Having two negative BCs or only one of two BCs growing a commensal prompts clinicians to discontinue empiric antibiotics [14].

In our study, pathogen distribution differed between single-culture and two-culture episodes. The most common pathogen was *Escherichia coli* in single-culture episodes and *Klebsiella pneumoniae* in two-culture episodes. This difference likely reflects the place of infection onset. Among episodes with a recorded admission date, 90% of single-culture episodes were taken during the first three days of hospitalization, while 48% of two-culture episodes were taken after day 3. In a nationwide study of Israeli adults, the predominant pathogen was *E. coli* in community-onset BSI and *K. pneumoniae* in hospital-onset BSI [32]. 

Our study’s implications for practice are that diagnostic stewardship interventions are needed to improve, taking two BC bottles in children when BSI is suspected and to minimize unnecessary BC collection. Several studies have evaluated interventions to lower unnecessary BC collection in pediatric patients. Mullan at el. implemented a program that included ordering guidelines, empowering ED phlebotomists to question orders for BC that seemed unnecessary, and drawing BCs but holding specimens at the bedside for up to 4 h while determining whether sending them to the lab was warranted [30]. The proportion of pediatric ED patients getting a blood culture declined by 27%, with no change in the number of return ED visits because of missed BSI. Woods-Hill et al. performed an intervention in the PICU that involved a fever/sepsis screening checklist and a blood culture decision algorithm; BC collection decreased by 46% with no change in in-hospital mortality or readmission [33].

The strength of our study is that it is based on a large data set representing 97% of Israel’s acute care hospitals. Our study has several limitations. First, because laboratories do not routinely record data on blood volume, we did not analyze this important variable that affects BC positivity. However, as we noted in the introduction, blood volume collected per bottle is often insufficient. Moreover, in our study, pediatric bottles were used in 88.9% of episodes, making blood volume insufficient for the majority of our sample. Therefore, either by increasing the volume or by increasing opportunities for detecting BSI, practically, drawing a second BC increased the detection rate. Second, we did not have clinical data and thus could not study clinical correlates of BC positivity. Third, we did not have data on antibiotic treatment prior to BC collection, which could affect bacterial growth. Fourth, the study is limited by its observational design; a randomized controlled trial would best determine whether two BCs are necessary in children.

In conclusion, when two BCs are collected, BSI is detected in a higher proportion of episodes. This reflects both improved sensitivity of the test and a higher pretest probability of BSI among children in whom two culture are taken. Thus, our study reveals two problems: under-detection of BSI by obtaining a single culture, and over-testing of patients with a low likelihood of BSI. Diagnostic stewardship interventions are required to address these two problems. Guidelines are needed that delineate the indications for obtaining BCs in children and advise taking two BCs when BCs are indicated. This is particularly important in the ED, where most BCs are drawn. 

## 4. Materials and Methods

### 4.1. Study Design and Data Collection

In this descriptive, retrospective study, we used data routinely collected from all Israeli hospitals as part of the Israeli Ministry of Health’s infection control program. Hospitals submit the following data on all BCs collected in a 6–10-month period each year: patient identification number, date of birth, admission date, ward, type of BC bottle (aerobic, anaerobic, or pediatric), the date and times of BC collection, and BC results. For this study, we used de-identified data from 2019–2021. 

### 4.2. Definitions and Inclusion Criteria

Patients under age 19 or hospitalized in pediatric wards (if birth date was not recorded) with at least one aerobic or pediatric BC were included in the study. We defined two BCs as two separate blood drawing occasions as indicated by separate specimen numbers and separate reporting in the laboratory report [29]. We defined a blood culturing episode as a 24-h period beginning with the first BC draw [10]. If blood draws continued for more than 24 h, we considered them part of the same episode until there were no blood draws for 24 h. For example, if blood samples for cultures were taken repeatedly over 72 h with no 24-h period without a blood draw, the 72 h were considered as one episode. The ward was defined as the first ward that drew the blood in each episode. We classified episodes into those with one BC or two, considering only aerobic or pediatric culture bottles. If more than two blood drawing occasions during an episode, we considered results only from the first two. We classified organisms as true pathogens or common commensals according to the National Healthcare Safety Network (NHSN) Organisms List [34]. Wards were classified as emergency department (ED), pediatric intensive care unit (PICU), neonatal intensive care unit (NICU), or other. We grouped patients into four age categories: birth to 1 month, 1 month to 1 year, 2–11 years, and 12–18 years. BCs drawn afterday 3 of hospitalization were considered as diagnosing possible nosocomial BSI.

### 4.3. Laboratory Methods

BCs were processed in the microbiology laboratories of the participating hospitals. All laboratories use automated blood culture systems, either BD BACTEC (Becton, Dickinson and Company, Franklin Lakes, NJ, USA), BACT/ALERT VIRTUO (bioMérieux, Lyon, France), or BACT/ALERT (bioMérieux). 

### 4.4. Statistical Analysis

We used chi-squared to test whether taking more than one BC varied by ward, age, or suspicion of nosocomial versus community-acquired BSI. We used a test of proportions to compare the true pathogen detection rate in episodes with one BC vs. two BCs. To determine whether the number of BCs taken per episode reflected confounding by indication (i.e., only one BC was taken because there was low suspicion of BSI), we used a test of proportions to compare the proportion of first BC with a true pathogen detected when additional BCs were taken versus when they were not. To estimate BSI that went undetected because only one BC was taken, we multiplied the proportion of episodes with a true pathogen detected only in second BCs by the number of negative single-culture episodes. Then, to account for the possibility of confounding by indication, we multiplied this result by the positivity ratio, i.e., the ratio of the proportion of first BC positive for a true pathogen when additional BCs were not taken versus when they were taken. Repeat positive blood culturing episodes (as defined above) in the same patient were included in all analyses except for the analyses of pathogen distribution. Analyses were done in Stata version 14.2 (Stata Corporation, College Station, TX, USA).

## Figures and Tables

**Table 1:** Ten most common true pathogens isolated in single-culture vs. two-culture blood culturing episodes in children.

Single-Culture Episodes		Two-Culture Episodes	
Pathogen	n (%)	Pathogen	n (%)
*Escherichia coli*	197 (11.3)	*Klebsiella pneumoniae*	272 (15.5)
*Staphylococcus aureus*	194 (11.2)	*Escherichia coli*	242 (13.8)
*Streptococcus pneumoniae*	138 (7.9)	*Staphylococcus aureus*	229 (13.0)
*Brucella* spp.	118 (6.8)	*Pseudomonas aeruginosa*	112 (6.4)
*Acinetobacter* spp.	107 (6.2)	*Enterobacter* spp.	87 (4.9)
*Klebsiella pneumoniae*	105 (6.0)	*Enterococcus faecalis*	78 (4.4)
*Streptococcus agalactiae*	69 (4.0)	*Streptococcus agalactiae*	68 (3.9)
*Haemophilus influenzae*	67 (3.9)	*Acinetobacter* spp.	66 (3.8)
*Pseudomonas aeruginosa*	64 (3.7)	*Candida* spp.	66 (3.8)
*Moraxella* spp.	64 (3.7)	*Streptococcus pneumoniae*	64 (3.6)
Other	615 (35.4)	Other	474 (27.0)
Total ^1^	1738 (100)	Total^1^	1758 (100)

^1^ Total is greater than the number of positive episodes because of polymicrobial infections.

**Table 2:** Characteristics associated with taking two blood cultures per culturing episode.

	1 Culture	2 Cultures	
	n (%)	n (%)	*p* ^1^
Full sample	160,964 (90.1)	17,738 (9.9)	
Ward			<0.001
Emergency department	85,213 (98.2)	1604 (1.8)	
Pediatric intensive care	7672 (70.0)	3281 (30.0)	
Neonatal intensive care	11,075 (86.5)	1727 (13.5)	
Other	57,004 (83.7)	11,126 (16.3)	
Age group			<0.001
<1 month	37,924 (88.5)	4925 (11.5)	
1–12 months	39,886 (93.4)	2833 (6.6)	
1–11 years	72,371 (90.6)	7535 (9.4)	
12–18 years	9983 (81.4)	2280 (18.6)	
Unknown	800 (82.9)	165 (17.1)	
Blood culture timing			<0.001
First 3 hospital days	109,374 (93.6)	7519 (6.4)	
After day 3	12,408 (64.3)	6885 (35.7)	
Unknown	39,182 (92.2)	3334 (7.8)	

^1^ Chi-squared test.

**Table 3:** True pathogen detection rate in one-culture vs. two-culture blood culturing episodes.

	1 Culture	2 Cultures
	n (%) with True Pathogen	n (%) with True Pathogen
Full sample	1687/160,964 (1.0)	1576/17,738 (8.9)
Ward		
Emergency department	685/85,213 (0.8)	199/1604 (12.4)
Pediatric intensive care	175/7672 (2.3)	353/3281 (10.8)
Neonatal intensive care	121/11,075 (1.1)	155/1727 (9.0)
Other	706/57,004 (1.2)	869/11,126 (7.8)
Age group		
<1 month	367/37,924 (1.0)	371/4925 (7.5)
1–12 months	427/39,886 (1.1)	363/2833 (12.8)
1–11 years	708/72,371 (1.0)	640/7535 (8.5)
12–18 years	176/9983 (1.8)	191/2280(8.4)
Unknown	9/800 (1.1)	11/165 (6.7)

Test of proportions *p* < 0.001 for full sample and for all wards and age groups.

## Data Availability

The data presented in this study are not publicly available due to privacy restrictions for healthcare data. Aggregate data will be made available upon justified request.
